# Sodium lignosulfonate improves shoot growth of *Oryza sativa* via enhancement of photosynthetic activity and reduced accumulation of reactive oxygen species

**DOI:** 10.1038/s41598-021-92401-x

**Published:** 2021-06-24

**Authors:** Andrew De-Xian Kok, Wan Muhamad Asrul Nizam Wan Abdullah, Chu-Nie Tang, Lee-Yoon Low, Mohd Hafis Yuswan, Janna Ong-Abdullah, Ngai-Paing Tan, Kok-Song Lai

**Affiliations:** 1 Department of Cell and Molecular Biology, Faculty of Biotechnology and Biomolecular Sciences, Universiti Putra Malaysia, 43400 UPM Serdang, Selangor Malaysia; 2 Laboratory of Halal Services, Halal Products Research Institute, Universiti Putra Malaysia, 43400 UPM Serdang, Selangor Malaysia; 3 Department of Land Management, Faculty of Agriculture, Universiti Putra Malaysia, 43400 UPM Serdang, Selangor Malaysia; 4 Health Sciences Division, Abu Dhabi Women’s College, Higher Colleges of Technology, 41012 Abu Dhabi, United Arab Emirates

**Keywords:** Plant biotechnology, Proteomics

## Abstract

Lignosulfonate (LS) is a by-product obtained during sulfite pulping process and is commonly used as a growth enhancer in plant growth. However, the underlying growth promoting mechanism of LS on shoot growth remains largely unknown. Hence, this study was undertaken to determine the potential application of eco-friendly ion-chelated LS complex [sodium LS (NaLS) and calcium LS (CaLS)] to enhance recalcitrant *indica* rice MR 219 shoot growth and to elucidate its underlying growth promoting mechanisms. In this study, the shoot apex of MR 219 rice was grown on Murashige and Skoog medium supplemented with different ion chelated LS complex (NaLS and CaLS) at 100, 200, 300 and 400 mg/L The NaLS was shown to be a better shoot growth enhancer as compared to CaLS, with optimum concentration of 300 mg/L. Subsequent comparative proteomic analysis revealed an increase of photosynthesis-related proteins [photosystem II (PSII) CP43 reaction center protein, photosystem I (PSI) iron-sulfur center, PSII CP47 reaction center protein, PSII protein D1], ribulose-1,5-bisphosphate carboxylase/oxygenase (Rubisco), carbohydrate metabolism-related proteins (glyceraldehyde-3-phosphate dehydrogenase 3, fructose-bisphosphate aldolase) and stress regulator proteins (peptide methionine sulfoxide reductase A4, delta-1-pyrroline-5-carboxylate synthase 1) abundance in NaLS-treated rice as compared to the control (MSO). Consistent with proteins detected, a significant increase in biochemical analyses involved in photosynthetic activities, carbohydrate metabolism and protein biosynthesis such as total chlorophyll, rubisco activity, total sugar and total protein contents were observed in NaLS-treated rice. This implies that NaLS plays a role in empowering photosynthesis activities that led to plant growth enhancement. In addition, the increased in abundance of stress regulator proteins were consistent with low levels of peroxidase activity, malondialdehyde content and phenylalanine ammonia lyase activity observed in NaLS-treated rice. These results suggest that NaLS plays a role in modulating cellular homeostasis to provide a conducive cellular environment for plant growth. Taken together, NaLS improved shoot growth of recalcitrant MR 219 rice by upregulation of photosynthetic activities and reduction of ROS accumulation leading to better plant growth.

## Introduction

Rice is an important food crop in the world, and it is a staple food for more than half the global population^1^. As the global population continues to grow, the demand in rice production increases as well. This eventually leads to an extensive usage of chemical fertilizers in order to meet with the increasing demand of the growing population. Based on the current population growth, it was expected that the global population to reach 9–11 billion by 2050^2^. In order to meet the global food demand by 2050, the agricultural produce is expected to grow by 70%^2^. However, current rice cultivation that relies heavily on chemical fertilizers has caused sustainability food production to be at stake due to deterioration of soil health and pollutions, which lead to loss of fertile land^3,4^. Approximately, one ton of rough rice production requires 20 kg of mineral nitrogen, 11 kg phosphorus oxide and 30 kg potassium oxide^5^. Hence, incorporation or replacement of current rice fertilization regime with environmental-friendly growth enhancer would be a promising alternative to sustain global rice production in order to meet the global food demand by 2050.

Lignosulfonate (LS) is a low-cost by-product obtained during sulfite pulping process. LS comprises of hydrophilic sulfonic groups and electroactive methoxyphenol, forming phenylpropane segments^6,7^. Similar to humic substances, LS contains high amount of carboxylic and phenol groups bound to aromatic rings, which enables chelation, buffering and cation exchange flexibilities^8^. In general, LS can be found chelated with different cations, such as calcium (Ca), sodium (Na), zinc (Zn), potassium (K) and iron (Fe), forming ion-chelated LS complex. The ability of LS to chelate different micronutrient ions enables it to be used as a plant fertilizer. In addition, LS chelator is more cost saving and eco-friendly as compared to other synthetic chelates, making it a good soil conditioners and plant stimulants in agriculture^9,10^. Therefore, application of LS in agriculture could potentially reduce the usage of chemical fertilizers.

Aside from agriculture application, several ion-chelated LS have also been used to enhance in vitro shoot and root growth of several plant species^8,11,12^. For instance, the incorporation of iron LS (FeLS) and calcium LS (CaLS) in medium successfully enhanced growth performance of holly, ginseng and poplar^13^. It was hypothesized that these ion-chelated LS are able to modulate the endogenous auxin and act as an auxin protector in order to prevent auxin degradation, which in turns resulted in vigorous root growth of the plants^13,14^. In addition, study by Docquier et al.^11^ suggested that incorporation of CaLS in the medium was able to enhance the nutrient ion availability to the plant, leading to better nutrient ion acquisition and growth performance of orchid, poplar and California redwood. A recent study made by Wan Abdullah et al.^15^ has successfully demonstrated that incorporation of CaLS is able to enhance callus growth in MR 219 rice via auxin biosynthesis and nutrient absorption. In another study by Wan Abdullah et al.^16^, they have also demonstrated that incorporation of CaLS improved the shoot proliferation rate of in vitro vanilla plant. The increased in vanilla shoot proliferation rate was associated with enhanced chlorophyll content, that contributed to the better photosynthetic activity for plant growth. Similarly, other humic substances like lignosulfonate-humate a, lignosulfonate-humate b and leonardite humic acid, have demonstrated to improve photosynthetic metabolism and stimulation of photosynthetic enzymes in maize^8^.

In plants, photosynthesis plays an essential role in plant growth and development, producing carbohydrates and energy required for plant growth. The regulation between photosynthesis and carbohydrate metabolism has been studied extensively. In general, plants utilized light energy to initiate electron transport chain producing NADPH and energy^17^. Subsequently, photosynthetic products produced were used for sugar production via Calvin cycle. The resulting sugar were then used to generate energy for plant growth and development through carbohydrate metabolism pathway^18^. However, during photosynthetic process, reactive oxygen species (ROS) were found to be constantly generated. ROS plays an important role in plant signaling^19^. However, constant generation of ROS may result in plant toxication which lead to detrimental effects to the plant growth. For instance, accumulation of ROS resulted in photoinhibition, leading to a reduction of photosynthetic activity^19^. Therefore, maintenance of optimum ROS level in the plant is vital in order to provide a conducive environment which able to stimulate their growth and development.

A recent study made by Wan Abdullah et al.^15^ described about the mechanism of CaLS involved during callus growth in MR 219 rice. However, this study only focused on the mechanism of ion-chelated LS complex during early plant development. The mechanism of ion-chelated LS complex is not only highly dependent on types of chelated ion and plant species, but it is also dependent on plant development stage^11,12^. Even though the incorporation of ion-chelated LS complex were shown to enhance shoot development in several plant studies, the mechanism involved during shoot development remains limited^11,13,16^.

Therefore, this study was undertaken to evaluate the mechanism of ion-chelated LS complexes, namely CaLS and sodium LS (NaLS), on shoot growth of recalcitrant *indica* rice cultivar. The mechanism underlying growth promoting effects of ion-chelated LS complex during shoot development were elucidated through label-free proteome profiling by studying the changes in protein abundance. Additionally, major biochemical analyses involved photosynthesis, carbohydrate metabolism and protein biosynthesis such as total chlorophyll content, rubisco activity, total sugar and total protein content were performed to provide an understanding on plant growth stimulation by LS. Besides, biochemical analyses involved in plant defense and stress response such as peroxidase activity, malondialdehyde content and phenylalanine ammonia lyase activity were performed in order to study the regulation of cellular homeostasis of the plant in response towards ion-chelated LS complex. Together with the current knowledge on the mechanism of LS, the results obtained in this study would provide a new insight on the mechanism underlying the growth promoting effects of LS during shoot development. Moreover, having a deeper understanding on the mechanism of LS would enable its usage efficiency to be maximized in plant tissue culture and agricultural sector, specifically in rice cultivation.

## Results

### Physiological responses of MR 219 rice shoot towards CaLS and NaLS treatments

**Figure 1:**
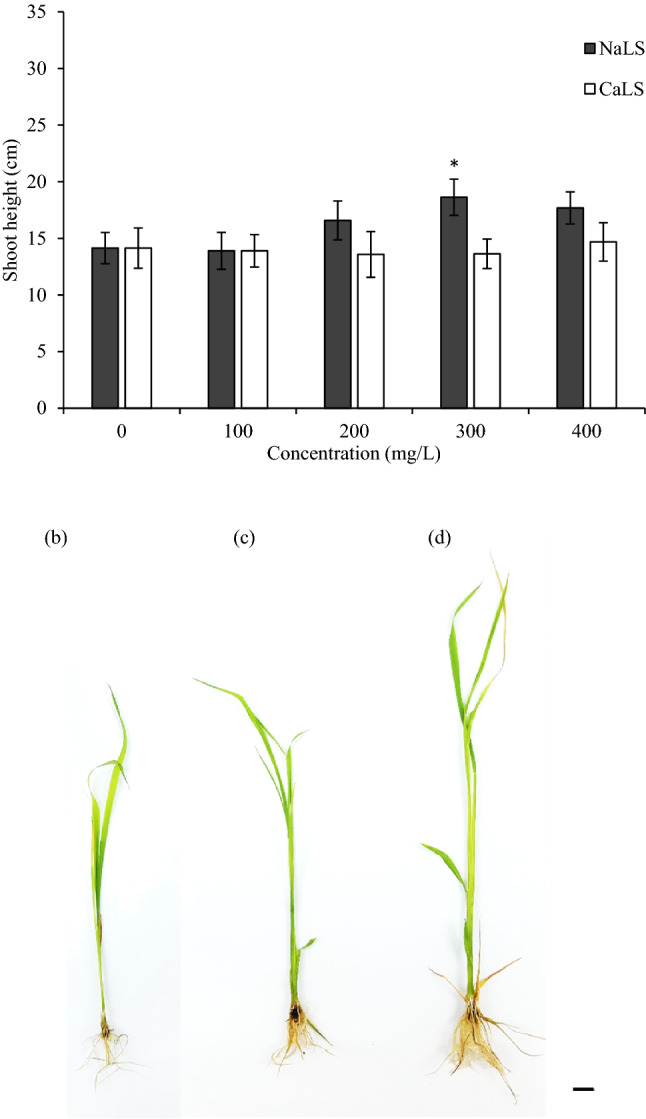
The MR 219 rice shoot growth in response to NaLS and CaLS treatments. (**a**) Shoot height after 3 weeks culture period in MSO, NaLS- and CaLS-supplemented medium. Phenotype of MR 219 rice showing the shoot growth after 3 weeks incubation on (**b**) MSO and (**c**) 300 mg/L CaLS- and (**d**) 300 mg/L NaLS-supplemented medium. Data shows mean of three biological replicates, n = 10, with error bars represent standard deviation. Asterisk represent the significantly difference between treatments at *p* < 0.05 when compared to MSO. Scale bars represent 1 cm.

Utilization of ion chelated LS required details optimization in order to provide beneficial effects to the plant. In this study, different concentrations of NaLS and CaLS (0, 100, 200, 300 and 400 mg/L) were supplemented into MS medium in order to determine their growth promoting effects during shoot growth of MR 219 rice. Shoot apex was selected in this study as it contained shoot apical meristem cells that contributed to building block of specialized tissues in the aerial parts of the test plant. In general, high concentration of NaLS (200 mg/L ≥) was found to enhance shoot elongation of the MR 219 rice (Fig. 1a). When compared to control plantlet (MSO; Fig. 1b), no significant changes were recorded with all concentrations tested for CaLS (Fig. 1a,c). On the other hand, the optimum concentration of NaLS was recorded at 300 mg/L, with significant increment in shoot growth (28.15%; Fig. 1d) as compared to MSO (Fig. 1a,b). Hence, further experiments were not performed on CaLS-treated plants. To understand the underlying mechanisms of NaLS in stimulating the shoot growth of rice, comparative proteome profiling and biochemical assays were performed on MSO and 300 mg/L NaLS plantlet (NaLS-treated rice).

### Comparative proteome analysis reveals an increased in photosynthesis-related proteins in NaLS-treated rice

Comparative proteome analysis was carried out to elucidate the changes in MR 219 rice proteome in response to NaLS in plant growth. The proteome analysis was performed using Perseus Software v1.6.0.7 (Max Planck Institute of Biochemistry, Planegg, Germany) to execute the Pearson correlation and Principal Component Analysis (PCA). Pearson correlation revealed strong positive correlation (> 0.7) between the MSO and NaLS-treated rice (Supplementary Fig. S1), which indicated that both MSO and NaLS-treated rice used were derived from the same organism with minimal contamination in the samples. In addition, the score plot of PCA showed grouping pattern of sample, wherein the MSO and NaLS-treated rice samples were totally separated because of significant change in proteome profile detected between treatment groups (Supplementary Fig. S1). Score plot of two principal components with PC1 and PC2 indicated 56.2% and 19.2%, respectively, thus made the total variance for the PCA is 75.4%. Meanwhile, the loading plot of PCA (Supplementary Fig. S1) describes the relationship among all proteins identified. These proteins identified in loading plot correlates to the samples as shown in score plot of PCA (Supplementary Fig. S1).

**Figure 2:**
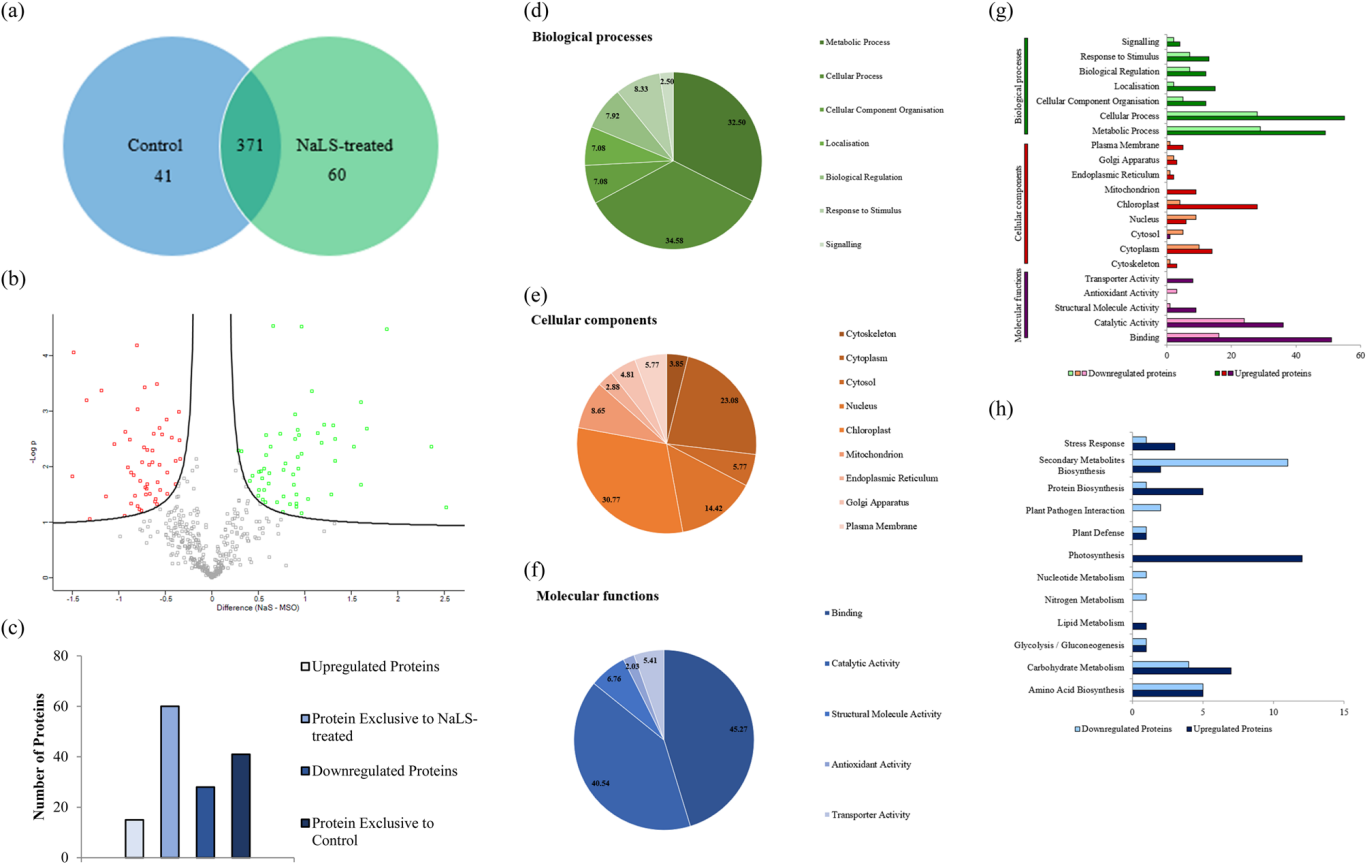
Comparative proteomic analysis between MSO and NaLS-treated rice. (**a**) Venn diagram of the total protein obtained from MSO and NaLS-treated rice. (**b**) Volcano plot showing up- (green squares) and downregulated (red squares) proteins of the NaLS-treated rice. (**c**) The total numbers of exclusive, up-regulated and down-regulated proteins in NaLS-treated rice compared to MSO. Gene ontology (GO) analysis in terms of (**d**) biological processes, (**e**) cellular components and (**f**) molecular functions of differentially expressed proteins and (**g**) their relative abundance of NaLS-treated rice. (**h**) KEGG pathway analysis of differentially expressed proteins found in NaLS-treated rice compared to MSO.

Based on comparative proteome analysis, a total of 41 and 60 unique proteins were successfully identified in the MSO and NaLS-treated rice, respectively; wherein, a total of 371 proteins were shared between the two samples (Fig. 2a). Through volcanic plot analysis, a total of 56 differentially expressed proteins were identified in response to NaLS. Among these proteins, a total of 15 proteins were upregulated, while a total of 41 proteins were downregulated in NaLS-treated rice as compared to the MSO plantlet (Fig. 2a–c). In response towards NaLS, proteins with the greatest increase in abundance were photosystem II (PSII) CP43 reaction center protein, photosystem I (PSI) iron-sulfur center, PSII CP47 reaction center protein and PSII protein D1. Meanwhile, proteins with greatest decrease in abundance were chitinase 5, chitinase 1, ATP synthase epsilon chain, thioredoxin reductase NTRB (Table 1). Two upregulated proteins [PSI iron-sulfur center (PSAC), PSII CP47 reaction center protein (PSBC)] and two downregulated proteins [chitinase 5 (CHT5) and chitinase 1 (CHT1)] were selected for gene expression analysis. Gene expression of selected proteins was observed to be in agreement with the proteome profile (Supplementary Fig. S2). The list of primers used in RT-qPCR can be found in Supplementary Table S2.

**Table 1:** Top 15 up- and down-regulated proteins of significant difference in NaLS-treated rice.

No.	Proteins	Uniprot accession no.	General function	Difference in protein abundance
**Upregulated proteins**				
1	Photosystem II CP43 protein	P0C367	Photosynthesis	1.1811
2	Photosystem I iron-sulfur center	P0C359	Photosynthesis	1.07513
3	Photosystem II CP47 protein	P0C362	Photosynthesis	0.962446
4	Photosystem II protein D1	P0C433	Photosynthesis	0.937846
5	Clathrin heavy chain 2	Q2QYW2	N/A	0.927112
6	Chlorophyll *a*/*b* binding protein, chloroplastic	A2XJ35	Photosynthesis	0.922371
7	Photosystem II D2 protein	P0C436	Photosynthesis	0.874711
8	Ribulose bisphosphate carboxylase small chain A, chloroplastic	P18566	Photosynthesis	0.66129
9	Chaperone protein ClpC2, chloroplastic	Q2QVG9	Translational modification	0.634115
10	Probable aldo-keto reductase 3	A2XRZ6	N/A	0.585803
11	Elongation factor 1-delta 2	Q40682	Protein biosynthesis	0.584471
12	Elongation factor 1-alpha	O64937	Protein biosynthesis	0.524749
13	Chaperone protein ClpC1, chloroplastic	Q7F9I1	Translational modification	0.504763
14	Glyceraldehyde-3-phosphate dehydrogenase 3, cytosolic	Q6K5G8	Carbohydrate metabolism	0.440004
15	Fructose-bisphosphate aldolase, chloroplastic	Q40677	Carbohydrate metabolism	0.322455
**Downregulated proteins**				
16	Chitinase 5	Q7Y1Z0	N/A	− 1.50325
17	Chitinase 1	Q42993	N/A	− 1.48961
18	ATP synthase epsilon chain, chloroplastic	P0C2Z1	N/A	− 1.34773
19	Thioredoxin reductase NTRB	Q6ZFU6	Amino acid biosynthesis	− 0.93522
20	Acyl transferase 9	Q9LGQ6	N/A	− 0.904535
21	Probable UDP-arabinopyranose mutase 2	Q7FAY6	Amino acid biosynthesis	− 0.885329
22	Ribose-phosphate pyrophosphokinase 4	Q6ZFT5	Amino acid biosynthesis	− 0.873207
23	Pyruvate kinase 1, cytosolic	B8BJ39	Glycolysis	− 0.842546
24	40S ribosomal protein S7	Q8LJU5	Translational modification	− 0.808968
25	Glucosidase 2 subunit beta	A2WNF5	Signaling and cellular process	− 0.803812
26	Cytochrome *c*	A2Y4S9	N/A	− 0.750532
27	Proteasome subunit alpha type-3	Q9LSU0	Protein folding	− 0.731038
28	Probable NADPH:quinone oxidoreductase 1	Q941Z0	N/A	− 0.723101
29	Glutaredoxin-C8	Q0DAE4	Protein folding	− 0.69478
30	Glutamine synthetase cytosolic isozyme 1-1	P14656	Nitrogen metabolism	− 0.640245

*N/A* not available.

The proteins identified were subjected to gene ontology (GO) analysis, in which they were classified into three categories, namely, biological processes, cellular components and molecular functions (Fig. 2d–f). From GO analysis (Fig. 2d–f) of biological processes, majority of the proteins were categorized under metabolic process (32.50%), followed by cellular process (34.58%), response to stimulus (8.33%) and biological regulation (7.92%). Meanwhile, when categorized under cellular components, majority of the proteins were involved in chloroplast (30.77%), cytoplasm (23.08%), nucleus (14.42%) and mitochondria (8.65%). In molecular functions, majority of the proteins identified were involved in binding (45.27%) and catalytic activity (40.54%). The changes in protein abundance in each category are shown in Fig. 2g. Subsequently, the identified proteins were subjected to KEGG pathway analysis^20^ in order to determine the overall effects of NaLS on the proteome during shoot growth (Fig. 2h). Based on KEGG pathway analysis^20^, proteins involved in secondary metabolite biosynthesis were found to be the most affected pathway in response towards NaLS, followed by photosynthesis, carbohydrate metabolism, and lastly, amino acid biosynthesis pathway (Fig. 2h). A detailed categorization of the proteome profile can be found in Supplementary Table S2.

### NaLS enhances protein synthesis and photosynthetic activities during shoot growth

**Figure 3:**
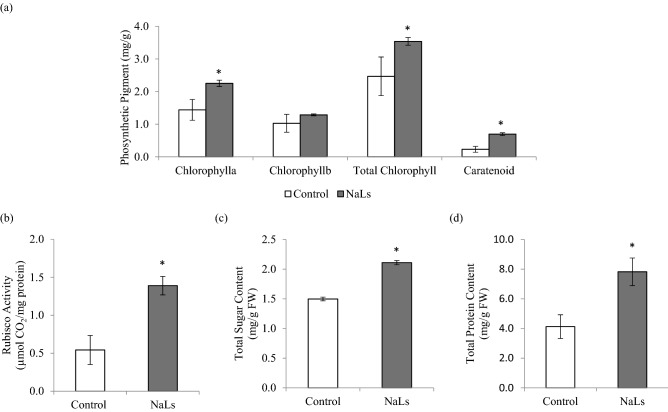
Evaluation of photosynthetic activity and protein biosynthesis related assays on MSO and NaLS-treated rice after 3 weeks incubation. (**a**) Total chlorophyll content; (**b**) rubisco activity; (**c**) total sugar content and (**d**) total protein content. Results show the mean of three biological replicates with error bars represent standard deviation. Asterisk indicates statistically significant difference at *p* < 0.05 when compared with MSO.

In addition to proteome analysis, biochemical assessments were performed to further elucidate the possible roles of NaLS during shoot growth. Our results in Fig. 3a recorded 3.54 mg/g fresh weight (FW) of total chlorophyll content in NaLS-treated rice as compared to the MSO (2.47 mg/g FW). The accumulation of chlorophyll content was coherent with the significant increase in rubisco activity, whereby significant increase in rubisco activity (1.39 µmol CO_2_/mg protein) was detected in NaLS-treated rice as compared to the MSO (0.54 µmol CO_2_/mg protein; Fig. 3b). Besides, significant increment of sugar content was also recorded in NaLS-treated rice (1.50 mg/g FW) as compared to the MSO (2.11 mg/g FW; Fig. 3c). Likewise, significant increment in protein contents were recorded in NaLS-treated rice (7.82 mg/g FW) as compared to the MSO (4.12 mg/g FW).

### Regulation of cellular homeostasis and stress response in NaLS-treated rice

**Figure 4:**
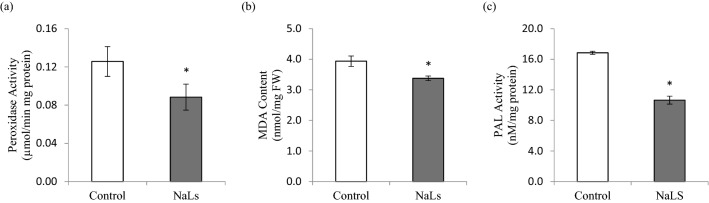
Stress related biochemical assays performed on MSO and NaLS-treated rice after 3 weeks incubation. Selected biochemical assays are (**a**) peroxidase activity; (**b**) malondialdehyde (MDA) content and (**c**) phenylalanine lyase (PAL) activity. Results show the mean of three biological replicates with error bars represent standard deviation. Asterisk indicates statistically significant difference at *p* < 0.05 when compared with MSO.

To investigate the possible involvement of secondary metabolites and stress response in NaLS-treated rice, peroxidase activity, malondialdehyde (MDA) content and phenyl ammonia lyase (PAL) activity were determined. The NaLS-treated rice recorded a significant decrease in peroxidase activity (0.08 µmol/min mg protein) as compared to MSO (0.13 µmol/min mg protein; Fig. 4a). Similar trend was observed in MDA content, in which a significant decrease was recorded in NaLS-treated rice (3.38 nmol/mg FW) when compared to the MSO (3.94 nmol/mg FW; Fig. 4b). As for PAL activity, a significant drop was also observed in NaLS-treated rice (16.84 nM/mg protein) as compared with the MSO (10.65 nM/mg protein; Fig. 4c).

## Discussion

Previous study has shown that beneficial effects of LS ion chelated complex is very much dependent on the type of tissues, plant species and type of chelated ions used. For instance, Docquier et al.^11^ reported the usage of CaLS (1000 mg/L) resulted in superior growth promoting effects compared to FeLS and potassium LS (KLS) in both *Phalaenopsis* and *Sequoia sempervirens*. In another study made by Kevers et al.^13^, a multiplication rate and vigor improvement of a shoot-proliferating poplar cluster were demonstrated after addition of FeLS and CaLS (10 mg/L). Meanwhile, in *Vanilla planifolia*, optimum shoot multiplication response was observed when supplemented with CaLS and NaLS (150 mg/L)^16^. Based on these studies, different concentrations and different types of ion-chelated LS complex were seen to enhance growth in various plant species. Hence, proper optimization on type of ion chelated and concentration of LS are required prior to its application in agriculture.

A recent study made by Wan Abdullah et al.^15^ reported the incorporation of CaLS (100 mg/L) resulted in optimum callus growth in *O. sativa* cv. MR 219. Wan Abdullah et al.^15^ has demonstrated that incorporation of CaLS promotes callus growth and root formation via enhanced endogenous auxin biosynthesis, nutrient uptake and regulation of cellular homeostasis^15^. However, in this study, NaLS was shown to be a better additive to improve the shoot growth of MR 219 rice when compared to CaLS. In this study, incorporation of NaLS were demonstrated to improve shoot development of MR 219 rice via stimulation of photosynthetic activities, carbohydrate metabolism and reduced accumulation of ROS. Based on these data, it is suggested that different ion-chelated LS may express different mode of action when introduced to different plant development stages^10^. Similar finding has also been reported by Low et al.^12^, whereby application of CaLS modulates different physiological response in different plant development stages of rice. In that study, application of CaLS observed an enhanced callus proliferation rate and adventitious root formation. However, application of CaLS was found to induce albino shoot formation during shoot induction study^12^. This indicate that a different mode of action of LS was expressed during shoot induction.

Incorporation of NaLS were observed to be the superior growth additive in enhancing shoot development when compared to CaLS. Although many studies have regarded that sodium ions (Na^+^) as non-essential element in terrestrial plant, their benefits in stimulating plant growth should not be ignored. In optimum concentration, Na^+^ could aids in regulating leaf turgor pressure and chlorophyll concentrations resulting in an enhancement of overall plant photosynthetic activity, which is essential for plant growth and development^21^. On top of that, Na^+^ could also be used as a substitute for K^+^, reducing K^+^ requirements for plant growth^22^. For instance, the germination rate, total dry weight and nutrient absorption of cotton plants were improved by Na supplementation^23^. Thus, application of NaLS increased the bioavailability of Na^+^ in the medium and enhanced uptake of Na^+^ in the plant, while reducing dependability towards K^+^ for plant growth.

On the other hand, incorporation of CaLS did not promote any shoot development in MR 219 rice. Similar finding was reported by Docquier et al.^11^, whereby supplementation of CaLS did not exhibit any effects on shoot multiplication in *S. sempervirens*. However, supplementation of CaLS (1000 mg/L) were reported to improve root growth of *S. sempervirens*. According to Docquier et al.^11^, the effects of LS could be indirect or ‘delayed’ and they hypothesized that the CaLS may either induce changes in hormonal, mineral balance, numbers of auxin receptor and/or their affinity towards free auxin.

Based on our proteomic data, increased of proteins abundance related to photosynthesis (PSII CP47, CP43, D1 and D2 proteins) was observed suggesting the participation of NaLS in governing photosynthetic rate. These proteins play an important role in modulation of photosynthetic activities in plant, as photosynthesis involves in production of energy and sugar required for plant growth. Moreover, previous study reported that moderate concentration of sodium chloride (up to 50 mM) allowed optimum Na^+^ accumulation on the leaves of *Zygophyllum xanthoxylum* which led to stimulation of photosynthesis activity and significantly increased relative growth rate^21^. Hence, NaLS may help in enhancing the photosynthetic rate to improve the rice growth as observed in this study.

Photosynthetic process is governed by a complex protein phosphorylation/dephosphorylation cascade^17^. The process is initiated by light-harvesting complexes, which absorb light and transfer excitation energy to the reaction centers of PSII and PSI. Subsequently, linear electron flow from PSII was initiated through a series of electron carrier intermediates producing and eventually reduced NADP to NADPH^24^. In present study, comparative proteome analysis reveals upregulation of photosynthesis-related proteins in response to NaLS (Table 1). The PSII is a multiprotein-pigment complex, composed of major protein subunits such as reaction center subunits (D1 and D2 proteins) and PSII internal antenna subunits (CP43 and CP47 proteins). According to Table 1, upregulation of PSII major protein subunits (PSII CP47, CP43, D1 and D2 proteins) were observed in NaLS-treated rice, suggesting an enhancement of PSII biogenesis. The PSII is one of the key components involved in initiation of linear electron flow of photosynthesis generating reducing power required for CO_2_ fixation to produce NADPH during photosynthesis^25^. Lacking in one of the major photosystem protein subunits may result in reduction of photosynthetic capacity and cellular metabolism. For instance, degradation of reaction center subunits (D1 and D2 proteins) was demonstrated to reduce photosynthetic performance in plants, which eventually reduced plant growth^18,25^.

In addition to proteome analysis, biochemical assessments were conducted to provide a further understanding on the possible roles of NaLS on shoot growth. Consistent with the upregulation of photosynthesis-related proteins in proteome profiling, the total chlorophyll content was significantly enhanced in response to NaLS treatment (Fig. 3a). The positive relationship between chlorophyll content and photosynthetic activity in plant has been widely observed^26^. In plants, chlorophyll is responsible for light-harvesting in photosynthesis, resulting in the excitation of electrons that are used to drive the linear electron flow in photosynthesis. Photosynthetic products produced will then be used for sugar production via Calvin cycle. In Calvin cycle, ribulose-1,5-bisphosphate carboxylase/oxygenase (Rubisco) plays an important role, whereby Rubisco catalyzes the assimilation of CO_2_ with ribulose-1,5-bisphosphate (RuBP) producing 3-phosphoglycerate (PGA). The PGA produced will be integrated into the Calvin cycle producing sugars^27^. In present study, the upregulation of Rubisco protein (Table 1) and enhanced Rubisco activity (Fig. 3b) were observed in NaLS-treated rice. Therefore, the increased in chlorophyll content accompanied with enhanced Rubisco activity support the role of NaLS in improving rice growth via enhanced photosynthetic activity.

Photosynthetic activity was known to influence sugar accumulation in plant. The data obtained in Fig. 3c shows that NaLS-treated rice had recorded significant amount of sugar content compared to the MSO. These observations were consistent with previous studies, in which enhanced photosynthetic activity were accompanied with enhanced sugar production^28,29^. On top of that, proteomic analysis revealed an upregulation of carbohydrate metabolism-related proteins [glyceraldehyde-3-phosphate dehydrogenase 3 (GAPDH) and fructose-bisphosphate aldolase (FBA); Table 1] in NaLS-treated rice. These two proteins were known to play a role in glycolysis pathway catalyzing the breakdown of glucose into pyruvate and energy, which will then be used for plant growth^30^. Likewise, NaLS-treated rice recorded a significant increase protein accumulation (Fig. 3d). The increased in protein content is consistent with the enhanced shoot growth and photosynthetic activity. Besides, studies have found positive correlation between protein content with nitrogen supply in plant^19,31^. Supporting this, it was demonstrated that deficiency in nitrogen supply in plant often reduces the growth of leaves by half. Nitrogen content is utilized as a source of building blocks for amino acid, which are prerequisite for protein biosynthesis in plant^19^. Besides, high protein content is crucial during plant growth and development as well. For instance, protein is involved as structural protein, transporter protein, and metabolic reaction in the cells^31^. These activities are important for proper functioning of the plant cells which will provide energy and food source to the plant and subsequently lead to improve plant growth.

Aside from photosynthetic process, NaLS was observed to be involved in modulation of ROS and stress in plant (Fig. 4 and Supplementary Table S1). During photosynthetic process, ROS were constantly generated. While ROS play an important role in plant signaling, excessive accumulation of ROS may cause oxidative damage towards plant cells, which leads metabolic homeostasis impairment. For instance, excessive ROS production caused by absorption of excessive sunlight by the light-harvesting complex resulted in photoinhibition of PSI^32^. Hence, it is vital to maintain cellular homeostasis in the plant during enhancement of photosynthetic activity. In present study, increased in protein abundance of peptide methionine sulfoxide reductase A4 (PMSRA4) and delta-1-pyrroline-5-carboxylate synthase 1 (P5CS1) that were found in NaLS-treated rice could act as stress regulator (Supplementary Table S1). Involvement of these proteins in regulating stress response in plant have been elucidated previously. As suggested by Romero et al.^33^, increased of PMSR4 activity could enhance tolerance level towards oxidative stresses. Similarly, P5CSI was shown to be vital as a stress regulator upon abiotic stress treatment.

Additionally, application of NaLS recorded a significant decrease in peroxidase activity (Fig. 4a). Low levels of peroxidase activity indicate low levels of ROS in plant cell. In line with the decrease in peroxidase activity, NaLS recorded a significant decrease in malondialdehyde (MDA) content (Fig. 4b). MDA is a major product of lipid peroxidation induced mainly by ROS and it reflects lipid peroxidation in plant cells in response towards stress^34^. Significant increase in MDA levels implies severe oxidative damage towards cell membrane. Studies have found that lipid peroxidation is a common phenomenon that occurs in plant cells when subjected to stress, and MDA are often used as a marker to determine the physiological status of the plant during plant growth^35^. On top of that, our study recorded a significant decrease of phenylalanine ammonia lyase (PAL) activity in NaLS-treated rice (Fig. 4c). The PAL is one of the rate-limiting enzymes, known to play a key role in secondary metabolite biosynthesis pathway. Secondary metabolites biosynthesis involves production of phenolic and flavonoid compounds which plays an important role in regulation of cellular homeostasis in plants in response towards stress^36,37^.

In general, regulation of cellular homeostasis is vital for enhancement of plant growth and development. During active acclimatization and adaptation process in response to stress environment, plant requires a bulk amount of energy for ATP biosynthesis and metabolism^38^. This could be seen during salt stress treatment of *Hordeum marinum,* whereby mitochondrial ATP synthase precursor and soluble inorganic pyrophosphatase 1 were upregulated to catalyze more energy to the plant indicating the urgent need for energy splurge in the form of ATP to establish homeostasis environment in the plant^39^. Therefore, the increase in abundance of stress regulator proteins, decrease in peroxidase activity, MDA content and PAL activity imply that incorporation of NaLS may play a role in reducing stress in plant cell. This allows the plant to be grown in a conducive environment, whereby energy produced by the plant could be used efficiently to promote plant growth and development rather than directed it for plant defense mechanism to maintain cellular homeostasis.

## Conclusions

**Figure 5:**
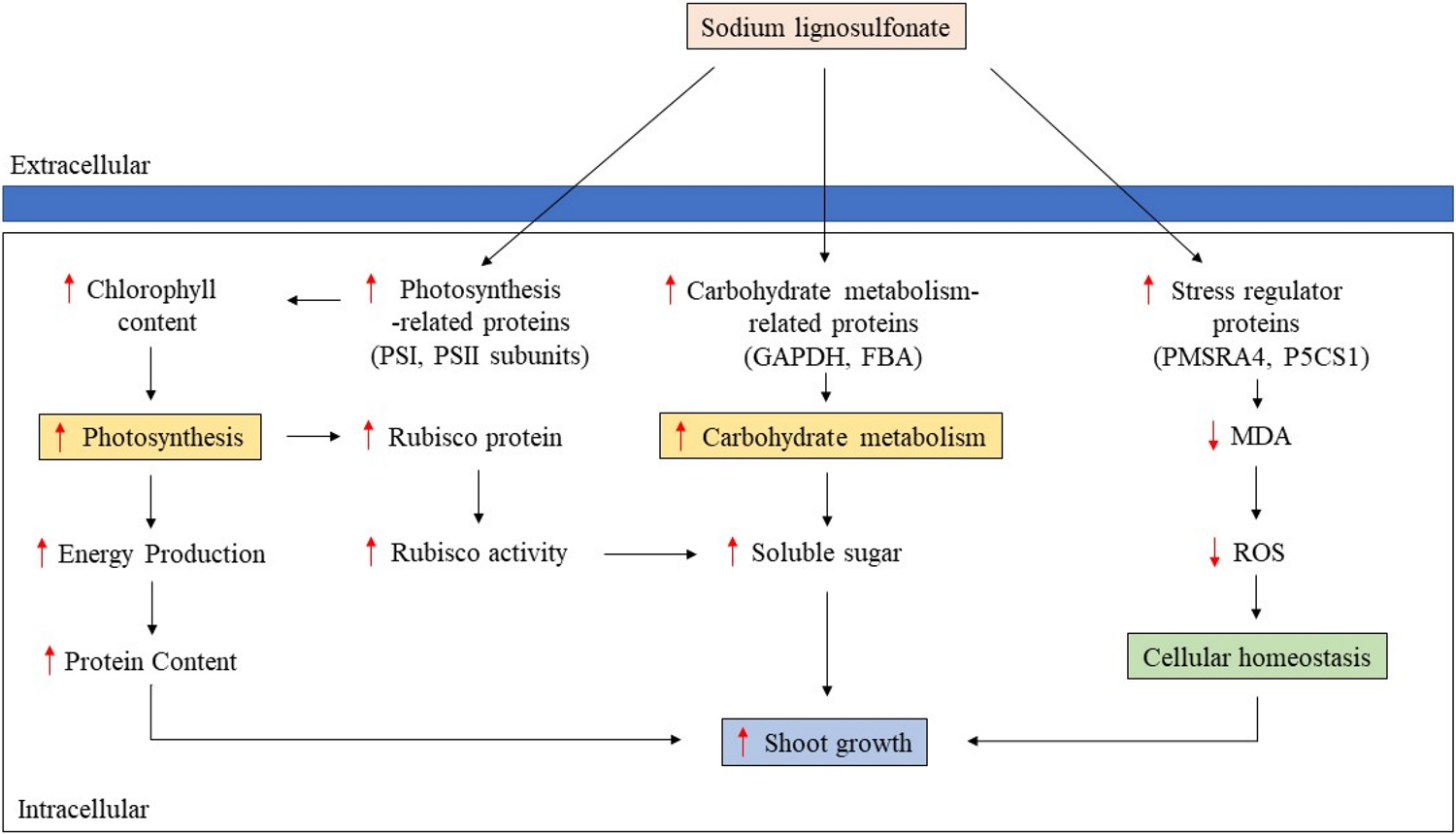
Proposed mode of action of NaLS on MR 219 during shoot growth. *PSI* photosystem I, *PSII* photosystem II, *GAPDH* glyceraldehyde-3-phosphate dehydrogenase 3, *FBA* fructose-bisphosphate aldolase, *PMSRA4* peptide methionine sulfoxide reductase A4, *P5CS1* delta-1-pyrroline-5-carboxylate synthase 1, *Rubisco* ribulose-1,5-bisphosphate carboxylase/oxygenase, *MDA* malondialdehyde, *ROS* reactive oxygen species, ↑ increased, ↓ decreased.

Our study demonstrated that the incorporation of 300 mg/L of NaLS in MS medium effectively enhanced shoot growth of MR 219 rice. Improvement of shoot growth was due to the stimulation of photosynthetic activities in the MR 219 rice, which could be evidenced by increased of PSI- and PSII- related proteins in proteome profiling as well as enhanced chlorophyll content and Rubisco activity in the plant (Fig. 5). In addition, it was found that NaLS successfully regulates cellular homeostasis in the plants, which was demonstrated by enhanced stress regulator proteins, reduced peroxidase activity, MDA content and PAL activity (Fig. 5). Hence, NaLS improved shoot growth of MR 219 rice via enhanced photosynthetic activities and ROS stabilization.

## Methods

### Plant materials

The seeds of the recalcitrant Malaysian rice cultivar MR 219 used in this research were obtained from Malaysian Agricultural Research and Development Institute (MARDI), Seberang Prai, Penang. We have received permission from MARDI for conducting research on MR 219 seeds. All the methodology and data collection comply with relevant institutional and national guidelines and legislation.

### Lignosulfonates preparation

Analytical grade NaLS (471038; Sigma-Aldrich, USA) and CaLS (471054; Sigma-Aldrich, USA) were prepared in a stock solutions of 50 mg/mL. All the stock solutions were filtered sterilized using 0.22 μm cellulose acetate membrane before being kept at 4 °C.

### Seed sterilization and LS treatment

Surface sterilization were performed on mature MR 219 seeds according to previously described protocol^40^ with slight modifications. Firstly, the seeds were de-husked and sterilized with 70% (v/v) ethanol for 1 min, followed by 50% (v/v) Clorox for 30 min. The seeds were rinsed with sterile distilled water before being dried on filter paper. The sterilized seeds were then transferred into our previously established callus induction medium containing Gamborg’s B5 basal medium^41^ supplemented with 10 g/L maltose, 0.1 g/L l-glutamine, 0.1 g/L l-asparagine, 0.1 g/L l-arginine, 10 mg/L NAA and 1 mg/L 2,4-D, pH 5.8^12^. The seeds were then incubated under a photoperiod of 16 h light and 8 h darkness at 25 ± 2 °C. After 1 week, approximately 1 cm of the shoot apices were removed from the seed and cultured in shoot growth medium containing Murashige and Skoog medium (MS)^42^ with 30 g/L sucrose, 3 mg/L kinetin and 0.5 mg/L NAA supplemented with CaLS or NaLS at different concentrations (100, 200, 300, and 400 mg/L). The shoot apices were incubated under a photoperiod of 16 h light and 8 h darkness at 25 ± 2 °C for 3 weeks. The experiment was then repeated twice (n = 10). All the estimations were performed in the 3 weeks old shoot of the test plant.

### Protein extraction and protein digestion

In proteomic analysis, plant samples were ground into fine powder using liquid nitrogen. The samples were then mixed with 500 μL protein extraction buffer containing 50 mM of ammonium bicarbonate and 10 mM phenylmethylsulfonyl fluoride (PMSF). The mixture was then vortexed, sonicated and centrifuged according to Yang et al.^43^ and solubilized protein was collected. Desalting was carried out on total soluble protein obtained using acetone precipitation method^44^. Subsequently, the proteins content in the plant sample was determined at 595 nm through Bradford assay^45^. The proteins sample (100 μg) was digested with Trypsin Gold (Promega, USA) at a ratio of 1:200 parts of protein, according to previous study^43^.

### Peptide separation and protein identification

Nano liquid chromatography tandem-mass spectrometry (nanoLC-MS/MS; Thermo Scientific, USA) analysis was performed according to Yang et al.^43^. Briefly, aliquot of 2 μL digested sample was injected into EASY-Spray Column Acclaim PepMap C18 100 (A0, 2 µm particle size, 50 µm id × 15 cm) at 35 °C. The sample elution process was performed similarly as described by Yang et al.^43^. The eluents from the LC were directly introduced into a mass spectrometer (Orbitrap Fusion—Thermo Fisher Scientific, US). The instrument was operated in the data dependent acquisition. Full scan spectra were collected (OTMS1) using parameters defined by previous study^43^. Only precursors with an assigned monoisotopic m/z and a charge state up to 4 were further analyzed for MS2. All precursors were filtered using a 20 s dynamic exclusion window and intensity threshold of 5000. The MS2 spectra were analyses (ITMS2) following parameters reported by Yang et al.^43^. Precursors were fragmented by CID and HCD at normalized collision energy of 30% and 28%, respectively.

Subsequently, raw data was analyzed using Thermo Scientific Proteome Discoverer Software 2.1 and SEQUEST HT was used as the database searching algorithm. The intensities of each MS ion were measured according to the accurate mass and time tag strategy^46^. Identification of the proteins was performed based on the searched against the UniprotKB database restricted to *O. sativa* (2020_01: 48,904 sequences) with a 1% strict FDR and 5% relax FDR criteria using Percolator. Search parameters were set up according to previous study^42^.

### Protein quantification and data analysis

The experiment was performed in triplicates with three biological replicates for MSO and NaLS-treated samples. The protein files (txt.format) obtained from Proteome Discoverer were uploaded to Perseus for comparative proteome analysis between MSO and NaLS-treated samples. Data processing such as PCA and Pearson correlation was performed in Perseus according to Ramdas et al.^46^. Significant differences of protein abundance were determined based on the Student’s *t* test (*p* < 0.05). The *p*-values were also adjusted for multiple-testing using the permutation-based false discovery rate, with a randomization number of 250. Proteins were considered to be significantly differentially expressed between treatment groups with adjusted *p*-values of < 0.05.

### Total photosynthetic pigments content

One hundred milligram of sample was ground into fine powder with the presence of liquid nitrogen. The ground powder was mixed with 2 mL of 80% (v/v) of acetone for 1 min in darkness. The homogenate was then centrifuged at 400×*g* for 5 min. The absorbance of the samples was then recorded at 470, 647 and 663 nm. The concentration of photosynthetic pigments (chlorophyll *a*, chlorophyll *b*, total chlorophyll and carotenoids) were calculated according to calculations described in Lichtenthaler et al.^47^ and expressed in mg/g fresh weight (FW).

### Rubisco activity

Rubisco activity was measured spectrophotometrically according to Usuda^48^ with slight modifications. In brief, 1 g of leaf sample was homogenized to fine powder with presence of liquid nitrogen. Then, the powder was mixed with ice-cold extraction buffer containing 0.25 M Tris–HCl (pH 7.8), 0.05 M MgCl_2_, 0.0025 M EDTA and 37.5 mg of DTT. Centrifugation of 10,000×*g* for 10 min at 4 °C was performed and the supernatant was collected as crude enzyme. Approximately 40 µL of crude enzyme was mixed with 960 µL reaction buffer containing 100 mM Tris–HCl (pH8), 40 mM NaHCO_3_, 10 mM MgCl_2_, 0.2 mM NADH, 4 mM ATP, 0.2 mM EDTA, 5 mM DTT, 1 U of glyceraldehyde-3-phosphodehydrogenase, 1 U of 3-phosphoglycerate kinase, and 0.2 mM ribulose 1,5-bisphosphate (RuBP). The absorbance of enzyme activities was recorded at 340 nm and expressed in µmol CO_2_ mg^−1^ protein.

### Total soluble sugar content

Total soluble sugar content was estimated according to Dubois et al.^49^ with slight modifications. In brief, 0.1 g of sample was ground into powder in liquid nitrogen and extracted twice in 2 mL of 90% (v/v) ethanol at 60 °C for one hour. After each extraction, samples were centrifuged at 400×*g* for 5 min. One mL of the supernatant was mixed with 1 mL of 5% (v/v) phenol together with 5 mL of concentrated sulphuric acid. The mixture was allowed to cool before the readings was taken at 495 nm spectrophotometrically. The soluble sugar content was determined using glucose as a standard and expressed in mg/g FW.

### Total protein content

Two hundred fifty milligram of plant sample were ground into powder with liquid nitrogen. Ice cold extraction buffer containing 1.8 mL of 50 mM ammonium bicarbonate (ABC) and 0.2 mL of 100 mM PMSF was added to the powdered sample. The homogenate was centrifuged at 10,000×*g* for 30 min and supernatant was collected as crude enzyme. All steps in enzyme extraction were performed at 4 °C. Bradford assay was performed to determine the protein concentration at 595 nm^45^. Total protein content in the sample was then determined by using bovine serum albumin as standard.

### Peroxidase activity

The crude enzyme obtained during protein extraction was used in determination of peroxidase activity. One hundred microliters of crude enzyme were added with 950 µL of distilled water, 750 µL of 100 mM potassium phosphate buffer (pH 6.8), 600 µL of 100 mM pyrogallol and 600 µL of 100 mM H_2_O_2_. Peroxidase activity were measured at 420 nm between the second and fifth minute after addition of crude enzyme into the substrate. The peroxidase activity was expressed as micromoles of purpurogallin formed per minute per milligram of proteins^50^.

### Malondialdehyde (MDA) content

MDA content was measured according to Luo et al.^51^ with slight modifications. Approximate 0.2 g of frozen powdered samples were dissolved in 10 mL of 10% (w/v) trichloroacetic acid (TCA). The homogenate was then centrifuged at 12,000×*g* for 10 min. Subsequently, 2 mL of supernatant was mixed with 2 mL of 10% (w/v) TCA containing 0.6% (w/v) of thiobarbituric acid (TBA) and incubated at 100 °C for 20 min. Then, the homogenate was allowed to cool on ice followed by centrifugation at 12,000×*g* for 10 min. The supernatant was then measured at 532, 600 and 450 nm. The MDA content was calculated using the following formula; MDA content (μM) = 6.45 (OD_532_ − OD_600_) − 0.56 (OD_450_).

### Phenylalanine ammonia-lyases (PAL) activity

PAL activity was measured according to Schmidt et al.^52^ with slight modifications. The crude enzyme obtained during protein extraction in total protein content determination was used to determine PAL activity in the sample. One hundred microliter of crude enzyme obtained were mixed with 1.15 mL containing 0.1 M sodium borate buffer (SBB; pH 8.8) and 10 mM l-phenylalanine. The mixture was then incubated at 37 °C for 1 h following which 250 µL of 5 N HCl was added to stop the reaction. PAL activity was then measured at 290 nm and the result was expressed in nanomoles of *trans-*cinnamic acid formed per milligram of proteins.

### Real-time reverse transcription polymerase chain reaction (RT-qPCR) analysis

The RNA was isolated via RNeasy Plant Mini Kit (Qiagen, Germany) following the protocol described in Lai^53^. First strand cDNA was converted from 1 μg of isolated total RNA using QuantiNova Reverse Transcription Kit (Qiagen, Germany). Expression profile were assessed via RT-qPCR analysis. Real-time PCR was performed with Bio-Rad CFX96 system (Bio-Rad, US) with QuantiNova SYBR Green PCR (Qiagen, Germany) following the protocol as described in Lai et al.^54^. The real-time PCR reaction conditions used were as follows: 95 °C for 30 s followed by 40 cycles of 95 °C for 5 s and 60 °C for 5 s. Three technical replicates on three biological replicates were performed on each sample. The data were analyzed using Bio-rad CFX Manager 3.1 software. The relative expression levels (2^−ΔΔCT^) were calculated according to Livak’s method^55^. The reference genes used in this study were rice *cyclophilin* (*OsCYC*) and *ubiquitin 5* (*OsUBQ5*).

### Statistical analysis

All data presented was the average ± standard deviation (SD) of three biological replicates. The Student’s *t* test was applied in evaluating the level of significant differences at *p* < 0.05 between the different treatments using the SPSS v.20 software (IBM Corp., Armonk, USA).

## Supplementary Information

Supplementary Information 1.

Supplementary Information 2.

Supplementary Information 3.

Supplementary Information 4.

Supplementary Information 5.
