# Supplementary material for: Sodium lignosulfonate improves shoot growth of Oryza sativa via enhancement of photosynthetic activity and reduced accumulation of reactive oxygen species
Source: Sci Rep. 2021 Jun 24;11:13226. doi: 10.1038/s41598-021-92401-x (PMC8225820; doi:10.1038/s41598-021-92401-x)
Supplement: Supplementary file 4 — Supplementary Information 4. [file 41598_2021_92401_MOESM4_ESM.docx]

**Article type: Original Research**

**Title: Sodium lignosulfonate improves shoot growth of *Oryza sativa* via enhancement of photosynthetic activity and reduced accumulation of reactive oxygen species**

Andrew De-Xian Kok^1^, Wan Muhamad Asrul Nizam Wan Abdullah^1^, Chu-Nie Tang^1^, Lee-Yoon Low^1^, Mohd Hafis Yuswan^2^, Janna Ong-Abdullah^1^ Ngai-Paing Tan^3^, & Kok-Song Lai^4^*

^1^Department of Cell and Molecular Biology, Faculty of Biotechnology and Biomolecular Sciences, Universiti Putra Malaysia, 43400 UPM Serdang, Selangor, Malaysia

^2^ Laboratory of Halal Services, Halal Products Research Institute, Universiti Putra Malaysia, 43400 UPM Serdang, Selangor, Malaysia

^3^ Department of Land Management, Faculty of Agriculture, Universiti Putra Malaysia, 43400 UPM Serdang, Selangor, Malaysia

^4^Health Sciences Division, Abu Dhabi Women’s College, Higher Colleges of Technology, 41012 Abu Dhabi, United Arab Emirates

*Corresponding authors: [lkoksong@hct.ac.ae](mailto:lkoksong@hct.ac.ae), [ngaipaing@upm.edu.my](mailto:ngaipaing@upm.edu.my)

**Supplementary Figure Legends**

**Supplementary Figure S1.** Exploratory analysis output of NaLS-treated rice via Perseus v1.6.0.7. (**a**) Score plot of Principal Component Analysis (PCA) attributes drastic variation between MSO (□) and NaLS-treated (□). (**b**) Loading plot of PCA. (**c**) Multi-scatter plot together with the Pearson correlation value reveals positive correlation between control and NaLS-treated rice.

**Supplementary Figure S2.** Normalized relative gene expression analysis of selected genes (*PSAC*, *PSBC*, *CHT5*, and *CHT1*) in MSO and NaLS-treated rice for proteomic validation. Data show the mean of three biological replicates. The selected genes were normalized with *OsEF1* and *OsUBQ5*. Asterisk indicates statistically significant difference at *p*<0.05 when compared to MSO. Error bars represent standard deviation.

**Supplementary Table Legends**

**Supplementary Table S1**. Proteins showing significant abundance difference (together with their accession numbers) in NaLS-treated rice. The list is sorted according to descending increased and decreased protein abundance of each protein.

**Supplementary Table S2** Primers used in RT-qPCR.

| (a) | (b) |
| --- | --- |
| 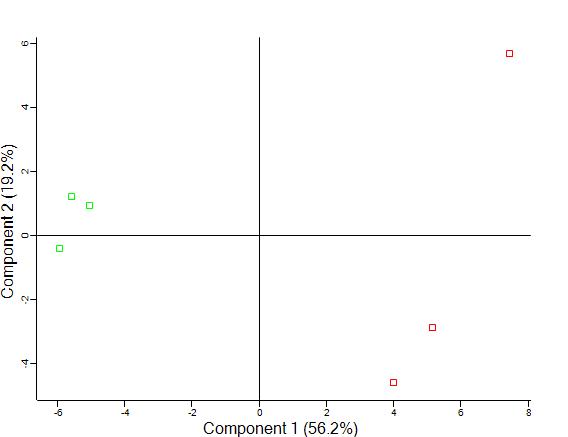 | 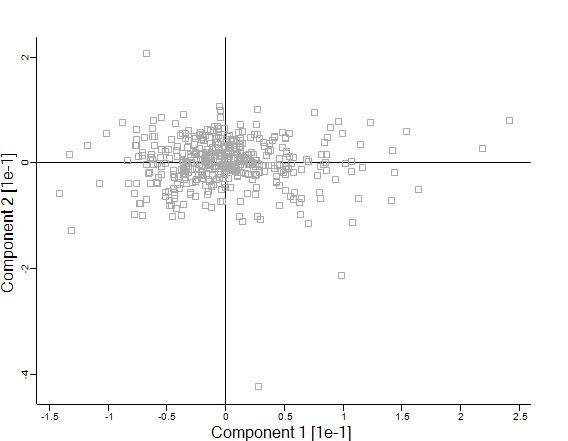 |
| (c) |  |
| 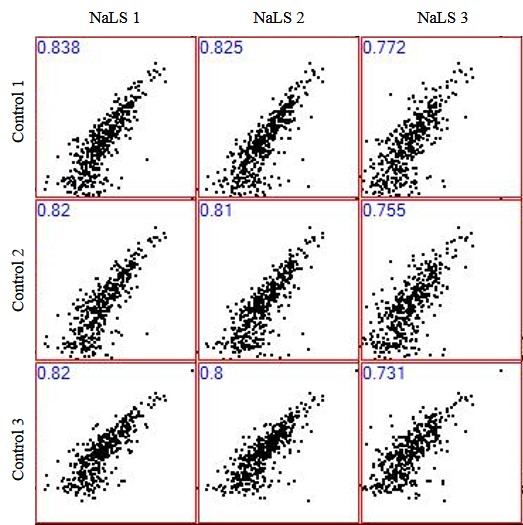 | |

**Supplementary Figure S1.** Exploratory analysis output of NaLS-treated rice via Perseus v1.6.0.7. (**a**) Score plot of Principal Component Analysis (PCA) attributes drastic variation between MSO (□) and NaLS-treated (□). (**b**) Loading plot of PCA. (**c**) Multi-scatter plot together with the Pearson correlation value reveals positive correlation between MSO and NaLS-treated rice.


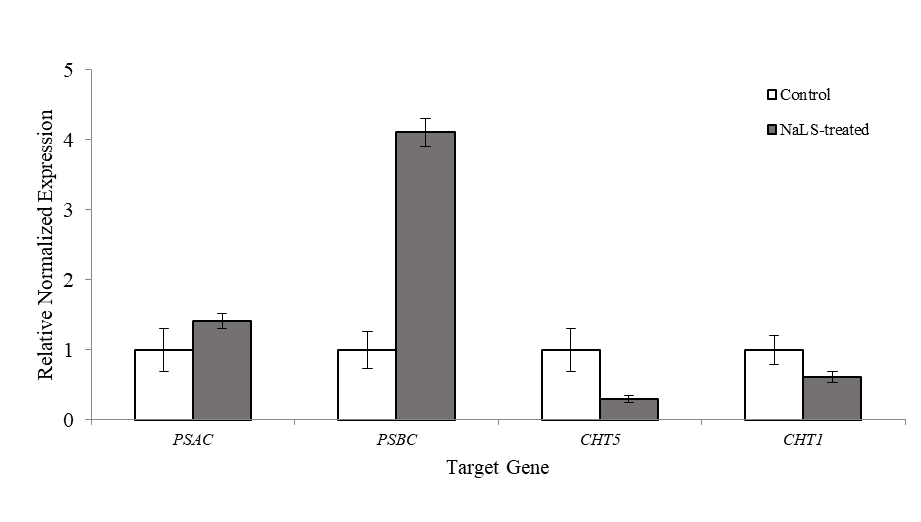


*

*

**Supplementary Figure S2.** Normalized relative gene expression analysis of selected genes (*PSAC*, *PSBC*, *CHT5*, and *CHT1*) in MSO and NaLS-treated rice for proteomic validation. Data show the mean of three biological replicates. The selected genes were normalized with *OsEF1* and *OsUBQ5*. Asterisk indicates statistically significant difference at *p*<0.05 when compared to MSO. Error bars represent standard deviation.

**Supplementary Table S1**. Proteins showing significant abundance difference (together with their accession numbers) in NaLS-treated rice. The list is sorted according to descending increased and decreased protein abundance of each protein.

| No | Proteins | | Uniprot Accession No. | General Function | Difference in Protein Abundance |
| --- | --- | --- | --- | --- | --- |
| **Upregulated proteins** | | | | | |
| 1 | Photosystem II CP43 protein |  | P0C367 | Photosynthesis | 1.1811 |
| 2 | Photosystem I iron-sulfur center |  | P0C359 | Photosynthesis | 1.07513 |
| 3 | Photosystem II CP47 protein |  | P0C362 | Photosynthesis | 0.962446 |
| 4 | Photosystem II D1 protein |  | P0C433 | Photosynthesis | 0.937846 |
| 5 | Clathrin heavy chain 2 |  | Q2QYW2 | Translational Modification | 0.927112 |
| 6 | Chlorophyll a-b binding protein, chloroplastic |  | A2XJ35 | Photosynthesis | 0.922371 |
| 7 | Photosystem II D2 protein |  | P0C436 | Photosynthesis | 0.874711 |
| 8 | Ribulose bisphosphate carboxylase small chain A, chloroplastic |  | P18566 | Photosynthesis | 0.66129 |
| 9 | Chaperone protein ClpC2, chloroplastic |  | Q2QVG9 | N/A | 0.634115 |
| 10 | Probable aldo-keto reductase 3 |  | A2XRZ6 | N/A | 0.585803 |
| 11 | Elongation factor 1-delta 2 |  | Q40682 | Protein Biosynthesis | 0.584471 |
| 12 | Elongation factor 1-alpha |  | O64937 | Protein Biosynthesis | 0.524749 |
| 13 | Chaperone protein ClpC1, chloroplastic |  | Q7F9I1 | Translational Modification | 0.504763 |
| 14 | Glyceraldehyde-3-phosphate dehydrogenase 3, cytosolic |  | Q6K5G8 | Carbohydrate Metabolism | 0.440004 |
| 15 | Fructose-bisphosphate aldolase, chloroplastic |  | Q40677 | Carbohydrate Metabolism | 0.322455 |
| **Proteins exclusive to NaLS-treated rice** | | | | | |
| 1 | ATP synthase subunit 9, mitochondrial |  | P0C519 | Energy Metabolism | NaLS-treated rice |
| 2 | ATP-dependent zinc metalloprotease FTSH 1, chloroplastic |  | Q5Z974 | Translational Modification | NaLS-treated rice |
| 3 | ATP-dependent zinc metalloprotease FTSH 2, chloroplastic |  | Q655S1 | Translational Modification | NaLS-treated rice |
| 4 | Carbamoyl-phosphate synthase small chain, chloroplastic |  | Q6YV23 | Amino Acid Metabolism | NaLS-treated rice |
| 5 | Chlorophyll a-b binding protein 1, chloroplastic |  | P12330 | Photosynthesis | NaLS-treated rice |
| 6 | Chlorophyll a-b binding protein 2, chloroplastic |  | P12331 | Photosynthesis | NaLS-treated rice |
| 7 | Cytochrome b6 |  | P0C315 | Photosynthesis | NaLS-treated rice |
| 8 | Delta-1-pyrroline-5-carboxylate synthase 1 |  | O04226 | Stress Response | NaLS-treated rice |
| 9 | Dolichyl-diphosphooligosaccharide--protein glycosyltransferase 48 kDa subunit |  | Q6ZLK0 | Translational Modification | NaLS-treated rice |
| 10 | Eukaryotic translation initiation factor isoform 4G-1 |  | Q84PB3 | Protein Biosynthesis | NaLS-treated rice |
| 11 | Glucose-1-phosphate adenylyltransferase large subunit 3, chloroplastic/amyloplastic |  | Q688T8 | Starch Biosynthesis | NaLS-treated rice |
| 12 | Glutamyl-tRNA(Gln) amidotransferase subunit B, chloroplastic/mitochondrial |  | Q2R2Z0 | Protein Biosynthesis | NaLS-treated rice |
| 13 | Leukotriene A-4 hydrolase homolog |  | Q84TA3 | Lipid Metabolism | NaLS-treated rice |
| 14 | Peptide methionine sulfoxide reductase A4, chloroplastic |  | Q336R9 | Stress Response | NaLS-treated rice |
| 15 | Photosystem I reaction center subunit VI, chloroplastic |  | Q0DG05 | Stress Response | NaLS-treated rice |
| **Downregulated proteins** | | | | | |
| 1 | Chitinase 5 |  | Q7Y1Z0 | N/A | -1.50325 |
| 2 | Chitinase 1 |  | Q42993 | N/A | -1.48961 |
| 3 | ATP synthase epsilon chain, chloroplastic |  | P0C2Z1 | N/A | -1.34773 |
| 4 | Thioredoxin reductase NTRB |  | Q6ZFU6 | Amino Acid Biosynthesis | -0.93522 |
| 5 | Acyl transferase 9 |  | Q9LGQ6 | N/A | -0.904535 |
| 6 | Probable UDP-arabinopyranose mutase 2 |  | Q7FAY6 | Amino Acid Metabolism | -0.885329 |
| 7 | Ribose-phosphate pyrophosphokinase 4 |  | Q6ZFT5 | Carbohydrate Metabolism | -0.873207 |
| 8 | Pyruvate kinase 1, cytosolic |  | B8BJ39 | Glycolysis | -0.842546 |
| 9 | 40S ribosomal protein S7 |  | Q8LJU5 | Translational Modification | -0.808968 |
| 10 | Glucosidase 2 subunit beta |  | A2WNF5 | Signalling and Cellular Process | -0.803812 |
| 11 | Cytochrome c |  | A2Y4S9 | N/A | -0.750532 |
| 12 | Proteasome subunit alpha type-3 |  | Q9LSU0 | Protein folding | -0.731038 |
| 13 | Probable NADPH:quinone oxidoreductase 1 |  | Q941Z0 | N/A | -0.723101 |
| 14 | Glutaredoxin-C8 |  | Q0DAE4 | Protein folding | -0.69478 |
| 15 | Glutamine synthetase cytosolic isozyme 1-1 |  | P14656 | Amino Acid Biosynthesis | -0.640245 |
| **Proteins exclusive to MSO** | | | | | |
| 1 | 24.1 kDa heat shock protein, mitochondrial |  | Q6Z7V2 | Stress Response | MSO |
| 2 | 2-C-methyl-D-erythritol 4-phosphate cytidylyltransferase, chloroplastic |  | Q5N8G1 | Secondary Metabolite Biosynthesis | MSO |
| 3 | 40S ribosomal protein S13-1 |  | Q69UI2 | Translation | MSO |
| 4 | 4-diphosphocytidyl-2-C-methyl-D-erythritol kinase, chloroplastic |  | Q8S2G0 | Secondary Metabolite Biosynthesis | MSO |
| 5 | 60S acidic ribosomal protein P3 |  | P56724 | Translation | MSO |
| 6 | Actin-3 |  | Q10AZ4 | Signalling and Cellular Process | MSO |
| 7 | Actin-7 |  | P0C540 | Signalling and Cellular Process | MSO |
| 8 | Aspartate carbamoyltransferase, chloroplastic |  | Q9LD61 | Amino Acid Biosynthesis | MSO |
| 9 | Calcium-dependent protein kinase 28 |  | Q2QX45 | Plant Pathogen Interaction | MSO |
| 10 | Serine/threonine-protein kinase SAPK5 |  | Q7XKA8 | Signalling and Cellular Process | MSO |
| 11 | Small ubiquitin-related modifier 1 |  | P55857 | Signalling and Cellular Process | MSO |
| 12 | Thioredoxin M2, chloroplastic |  | Q7X8R5 | Transporter Protein | MSO |
| 13 | Two-component response regulator ORR11 |  | Q6H468 | Signalling and Cellular Process | MSO |
| 14 | U2 small nuclear ribonucleoprotein B |  | Q6ETX3 | Translational Modification | MSO |
| 15 | UDP-arabinopyranose mutase 3 |  | Q6Z4G3 | Nucleotide Metabolism | MSO |

N/A, not available

**Supplementary Table S2** Primers used in RT-qPCR.

| Target genes | Primer Sequence 5`–3` | |
| --- | --- | --- |
| *PSAC* | Forward | ATGCTGCGGGTTGTCTCAGGTC |
|  | Reverse | ACCGAAGATTGTGTGGGTTGTAAG |
| *PSBC* | Forward | AGCTCACGTAGCCCATGCAG |
|  | Reverse | ATAAATGCCACCGAAGCCTAAGAC |
| *CHT1* | Forward | GTTCTGGTTCTGGATGACGC |
|  | Reverse | TGTAGAACCCAATCCGGTCG |
| *CHT5* | Forward | CAAGGACTACTGCGGAGATGG |
|  | Reverse | GACCACGCTCTCTACAGACAC |
| Housekeeping genes | Primer Sequence 5`–3` | |
| *EF1* | Forward | ACCAGATCAACGAGCCCAAG |
|  | Reverse | CTCCAGTCTCAACACGACCC |
| *UBQ5* | Forward | TAGGCGTAGGCTCCTGTTCT |
|  | Reverse | ACAGAGGTGATGCTAAGGTGT |
